# Terminal Ileitis as the Presenting Feature of Henoch-Schönlein Purpura in a 22-Year-Old Male

**DOI:** 10.7759/cureus.19406

**Published:** 2021-11-09

**Authors:** Muhammad Waleed, Swaminathan Perinkulam Sathyanarayanan, Soban Arif Maan, Linta Mansoor, Kayla Hoerschgen

**Affiliations:** 1 Internal Medicine, University of South Dakota Sanford School of Medicine, Sioux Falls, USA; 2 Internal Medicine: Diabetes and Endocrinology, Aga Khan University Hospital, Karachi, PAK; 3 Internal Medicine, Faisalabad Medical University, Faisalabad, PAK; 4 Pathology, University of South Dakota Sanford School of Medicine, Sioux Falls, USA

**Keywords:** henoch-schönlein purpura, gastrointestinal bleed, iga nephritis, henoch-schönlein purpura (iga vasculitis), terminal ileitis

## Abstract

Henoch-Schönlein purpura (HSP) is a self-limited vasculitis that affects children and the preadolescent population. It is characterized by the deposition of immunoglobulin A immune complexes in tissues leading to palpable purpura, abdominal pain, arthritis, and nephropathy. When it occurs in adults, the clinical manifestations are the same; however, adults present with more significant renal involvement. While abdominal pain is the most common gastrointestinal (GI) manifestation, it can also present with GI bleeding, intussusception, bowel ischemia, and bowel perforation. Here, we report the case of a 22-year-old gentleman who presented with nonspecific GI complaints such as nausea, vomiting, and loose stools. He was later found to have terminal ileitis preceding the onset of rash, the biopsy of which confirmed HSP. Terminal ileitis is a rare GI manifestation of HSP and is not very commonly reported in the literature.

## Introduction

Henoch-Schönlein purpura (HSP), also known as immunoglobulin A (IgA) vasculitis, is typically a self-limited autoimmune disease. Although rare in adults, it is the most common childhood vasculitis affecting 10 to 20 children per 100,000 every year and an approximate annual prevalence of 13 to 15 cases per 1,000,000 in adults [1-5]. Upper respiratory tract infections and/or intake of certain drugs are considered precipitating factors for HSP; however, its exact etiology remains unknown. It is a small vessel vasculitis caused by antigen-antibody (IgA) immune complex deposition which leads to inflammation, characteristically manifesting as a palpable purpuric rash on the buttocks and lower extremities [6]. Multiple other organs are also involved, with renal dysfunction, joint involvement, and gastrointestinal (GI) symptoms being the most frequent ones. Abdominal pain is the primary presentation of GI involvement, along with other nonspecific symptoms such as nausea, vomiting, and diarrhea [2].

Terminal ileitis associated with HSP has rarely been reported in the medical literature. Here, we present an unusual case in which a patient presenting with abdominal pain preceding a rash was diagnosed with terminal ileitis on colonoscopy, and later a skin biopsy confirmed HSP.

## Case presentation

**Figure 1:**
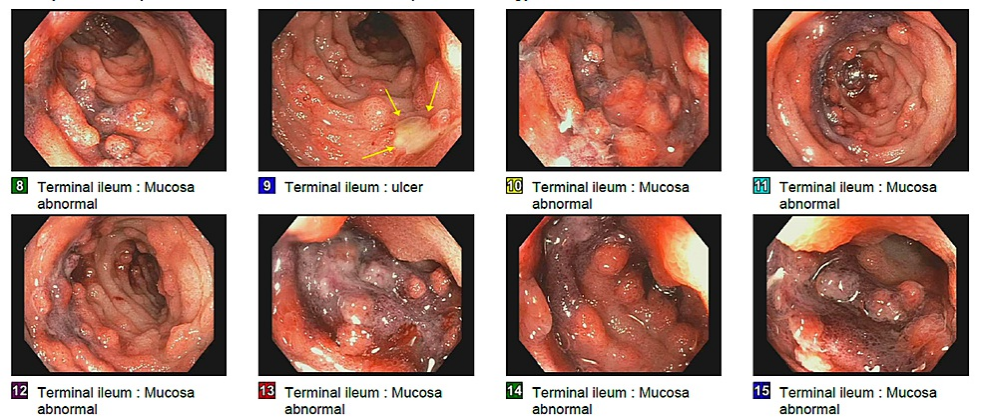
Colonoscopy showing features of terminal ileitis.

A 22-year-old Caucasian gentleman with no prior medical history presented to the emergency room with complaints of severe lower abdominal pain, nausea, vomiting, and diarrhea. He denied fevers, chills, joint pains, skin rashes, and blood in the stools at that time. He was a lifelong nonsmoker, consumed one to two alcoholic drinks per week, denied substance abuse, and was monogamous with a female partner. He had no significant family history. His vital signs were unremarkable. Physical examination showed tenderness in the lower quadrants of his abdomen. His initial labs showed a leukocyte count of 15.9 k/uL, but otherwise unremarkable complete blood count, renal function tests, and liver function tests. Computed tomography (CT) of the abdomen/pelvis showed wall thickening of the small intestine. Given the acuteness of the onset of his symptoms, an infectious etiology was suspected, and he was empirically started on levofloxacin and metronidazole. A comprehensive enteric panel to rule out infectious colitis was negative. His symptoms worsened with the development of blood-streaked emesis and hematochezia, and his labs showed an increase in leukocyte count to 21 k/uL; however, his hemoglobin remained stable. Thus, gastroenterology evaluation was sought. His stool calprotectin levels were elevated at 220 µg/g (reference range: <50 µg/g) and C-reactive protein was elevated at 58 mg/L (reference range: <5 mg/L). He underwent upper endoscopy and colonoscopy. Upper endoscopy was unremarkable, but colonoscopy showed areas of granular, erythematous, ulcerated, hemorrhagic, and ecchymotic mucosa in the terminal ileum (Figure 1). His colonic mucosa appeared relatively normal. There was also blood in the terminal ileum and the colon. However, no discrete areas of bleeding were identified. These findings were consistent with moderate-to-severe active terminal ileitis, raising the suspicion for Crohn’s disease.

**Figure 2:**
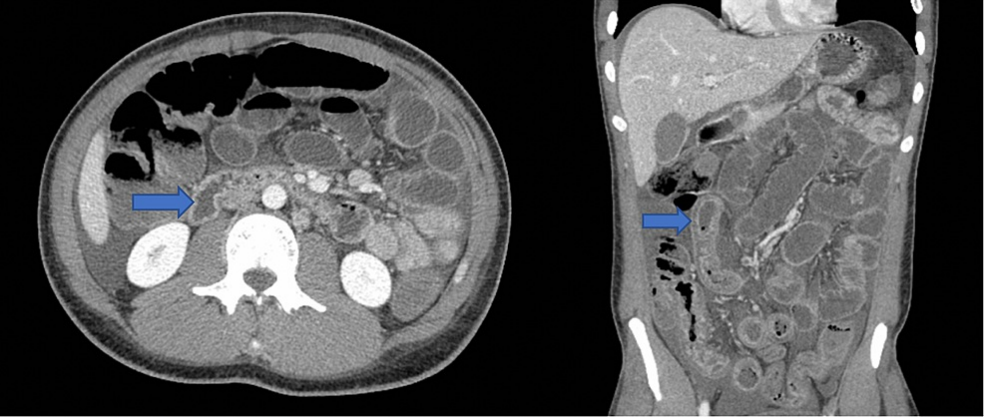
Small bowel thickening and narrowing of the terminal ileum (blue arrows).

**Figure 3:**
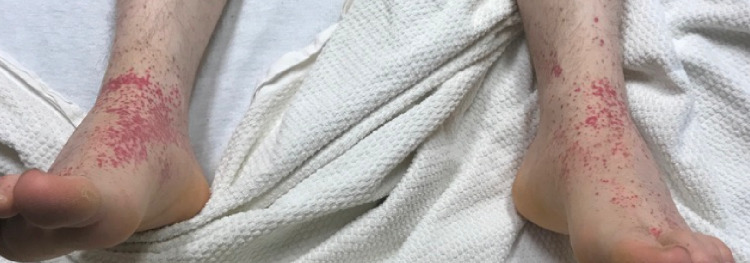
Palpable purpuric rash.

CT enterography (Figure 2) was also obtained to further investigate the small bowel which showed small bowel inflammatory changes in the ileum, supporting the suspicion for Crohn’s disease. However, while awaiting his ileal biopsy reports, his symptoms evolved and he developed bilateral knee pain and nonblanching, erythematous, coalescing, palpable, maculopapular rash on the dorsal aspect of the feet and around the ankles (Figure 3). Further workup with antinuclear antibodies, antineutrophil cytoplasmic antibodies, and complement levels C3 and C4 was unremarkable. He tested positive for *Chlamydia trachomatis* in his urine. However, he did not have features of dactylitis, conjunctivitis, or enthesitis nor did he have any symptoms of chlamydial urethritis such as urethral discharge prior to the onset of his symptoms, making reactive arthritis unlikely as the cause of his new-onset arthralgias.

**Figure 4:**
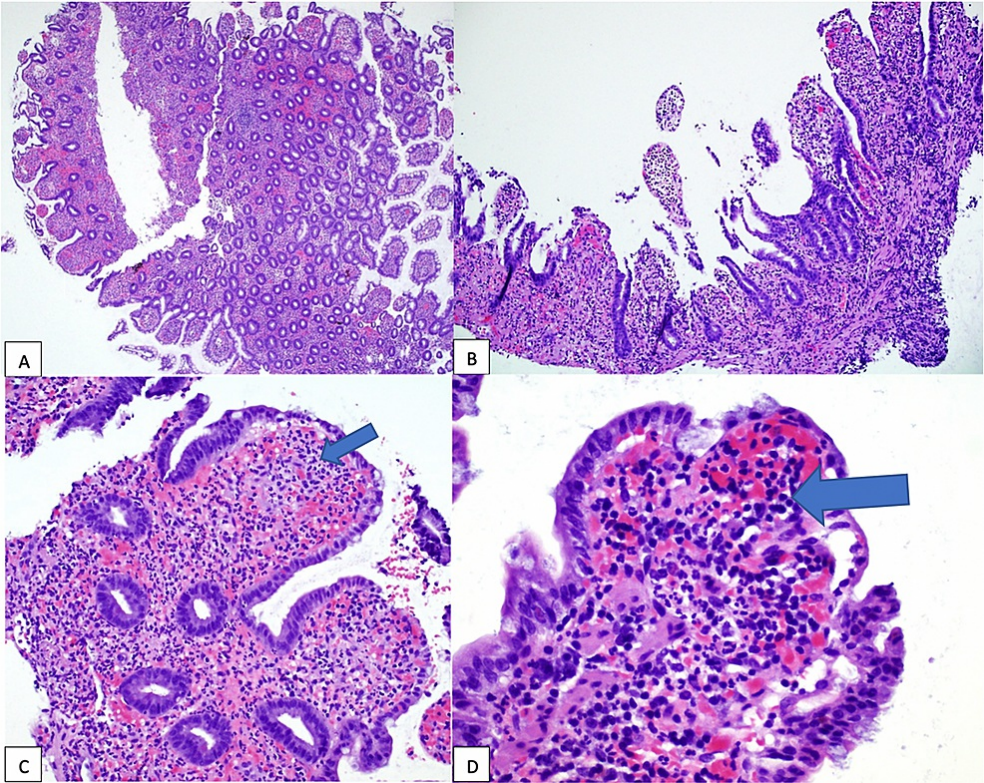
Biopsies of the terminal ileum. (A) Ileal mucosa with increased inflammation in the lamina propria and blunted villi (on the left). (B) Ulceration of ileal mucosa with no identifiable villi in the center. (C) Blunted villi with neutrophils (arrow) in the lamina propria and mucosa. (D) Villi with a prominent neutrophilic infiltrate (arrow) in the lamina propria and mucosa (hematoxylin and eosin, original magnifications ×40 [A], ×100 [B], ×200 [C and D]).

**Figure 5:**
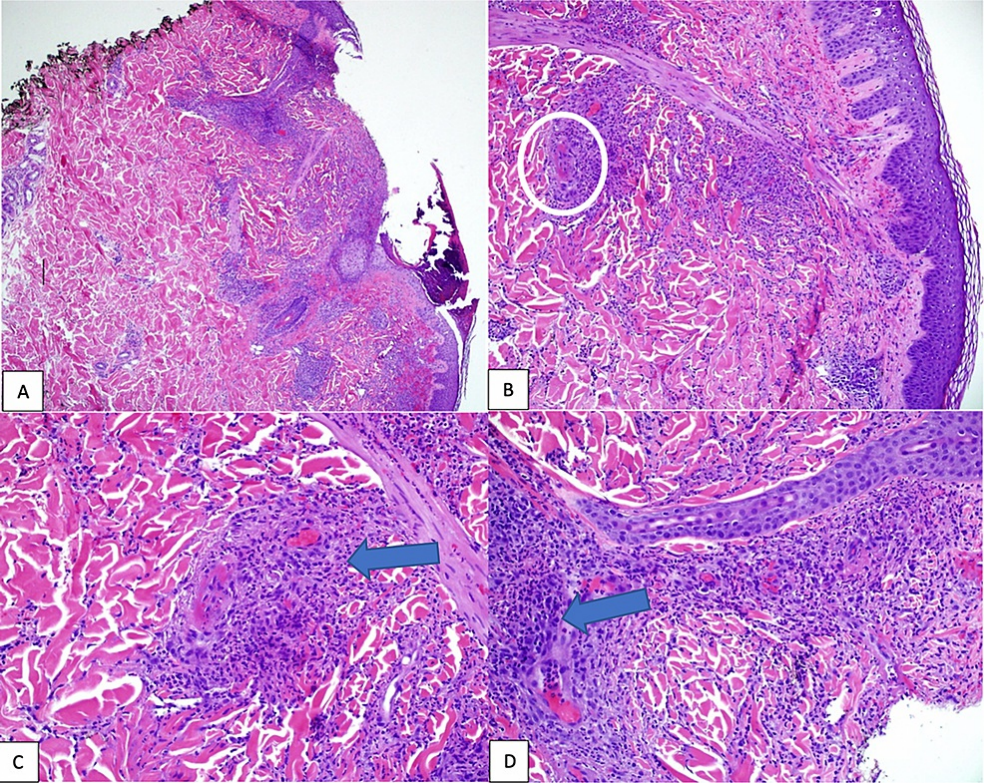
Punch biopsy of the right lateral calf. (A) Excoriation of the epidermis (right) with underlying acute and chronic inflammation surrounding adnexal structures and blood vessels. (B) Superficial perivascular infiltrate of neutrophils with fibrin deposition within the blood vessel (circle). (C) Neutrophils surrounding a vessel with fibrin deposition (arrow). (D) Acute and chronic inflammation surrounding vessels and infiltrating dermal collagen bundles (arrow) (hematoxylin and eosin, original magnifications ×40 [A], ×100 [B], ×200 [C and D]).

His ileal biopsy report showed moderate-to-severe active ileitis, with ileal ulceration and neutrophil deposition in the lamina propria and mucosa (Figure 4). However, there were no noncaseating granulomas or chronic inflammatory cell deposition, which are characteristically seen in Crohn’s disease. Moreover, with new arthralgias, nonpalpable purpura on top of enteritis, HSP was suspected. Thus, a skin biopsy of the rash was obtained and he was also started on intravenous methylprednisolone 40 mg daily and 1 g azithromycin one time for chlamydia. Skin biopsy demonstrated HSP (Figure 5). His GI symptoms improved dramatically after initiating steroids. His renal function was monitored throughout the hospital stay. His renal function remained stable, and his urinalysis did not show any evidence of hematuria or proteinuria. Prior to discharge, he was seen by rheumatology who recommended sending him home on a course of prednisone taper with close outpatient follow-up. On follow-up visits with rheumatology, his symptoms improved significantly, and his steroids were slowly tapered off. He was also seen in the GI clinic after four months of completion of steroid therapy, at which time he had no abdominal pain or diarrhea. His stool calprotectin levels were also normalized at that time.

## Discussion

HSP is characterized by the presence of palpable purpura without thrombocytopenia or coagulopathy and at least one of the following: arthritis/arthralgia, abdominal pain, leukocytoclastic vasculitis/IgA deposition, and nephritis [7]. It is a self-limiting disease with a higher incidence in children (over 90%), occurring less commonly in adults. Adult populations tend to have a more severe clinical picture with a significantly higher incidence of GI bleed and nephropathy, and renal involvement in adults accounts for a poor prognosis [1,3]. Diagnosing HSP can be very challenging when cutaneous manifestations present later during the clinical course following the onset of abdominal pain or arthralgias. Skin rash is the most commonly occurring symptom in both children and adults, although not necessarily in the early stages of the disease [3]. In an epidemiological study involving 78 children with HSP conducted by Calvino et al. [2], while all children developed nonthrombocytopenic palpable purpura during the course of the disease, other system manifestations such as abdominal pain and/or joint manifestations preceded the onset of purpura in almost 31% of the patients.

The most common GI manifestation in HSP is colicky abdominal pain secondary to peritoneal and visceral purpura resulting in mucosal and submucosal extravasation of blood and edema fluid, further complicating into ulceration and GI bleeding. Other uncommon complications include intussusception, malabsorption, hemorrhagic ascites, perforation, and even strictures [2,8]. Occurrence of abdominal symptoms is reported in 80-85% of patients. While HSP affects the small bowel predominantly, it can also involve the esophagus, stomach, terminal ileum, and colon [9]. There have been reports of mild liver damage and cholestasis, albeit for a brief period of time [5]. In a study by Pillebout et al. [5] of HSP in adults, GI involvement was seen in 48% of the population. GI involvement as the presenting complaint was seen in 8% of the study population.

Radiologically, GI involvement in HSP presents as areas of bowel thickening with skip lesions, symmetry of small bowel folds due to edema, mesenteric edema, vascular engorgement, intramural and extramural masses, and nonspecific lymphadenopathy [8,9]. Endoscopic features of HSP include petechiae, erythema, edema, ulcers, nodules, hematoma-like protrusions, and strictures [10]. An observational study was conducted by Louie et al. [11] which involved an analysis of GI biopsy samples of 16 patients with HSP (both children and adults). All cases had lamina propria hemorrhage with several reports of lamina propria fibrin deposition. Overall, twelve of sixteen duodenal biopsies showed duodenitis, seven out of nine jejunal and ileal biopsies showed jejunitis/ileitis, and nine of twelve colorectal biopsies had colitis, demonstrating that most patients have inflammatory cell deposits in the GI tract.

Terminal ileitis as a GI manifestation of HSP is a rarely reported phenomenon in the literature. Terminal ileitis has been known as the pathognomonic hallmark of Crohn’s disease. When patients present with HSP accompanied by terminal ileitis, the possible differential diagnoses to be sought include Crohn’s disease, vasculitides, and infections [12]. The resemblance of the inflammatory lesions in the terminal ileum between HSP and Crohn’s disease hints at a potential pathophysiological link between the two disorders, which is supported by an emerging body of evidence in the literature. There have been instances of occurrence of Crohn’s disease later in life after an episode of HSP, occurrences of HSP in those with Crohn’s disease treated with adalimumab, and incidence of HSP in children of a mother with Crohn’s disease [7]. Additionally, there are also reports of HSP with terminal ileitis complicated by thrombotic microangiopathy [13], as well as in association with rapidly progressing glomerulonephritis triggered by pantoprazole [14].

HSP is self-limiting in almost 90% of all patients. Systemic steroids should be used in those with severe rash, edema, severe abdominal pain, and renal involvement. Treatment includes prednisone or methylprednisolone 1 to 2 mg/kg for one to two weeks followed by a taper to 0.5 mg/kg/day over the next week and then 0.5 mg/kg/day every other day for another week. Steroids help prevent major complications such as severe GI bleeding and intussusception [10].

## Conclusions

This case report highlights a rare presentation of HSP with terminal ileitis as the initial presenting feature preceding skin manifestations.
